# An overview of the heat-induced changes of the chemical composition of bone from fresh to calcined

**DOI:** 10.1007/s00414-024-03160-z

**Published:** 2024-01-25

**Authors:** Thomas P. Shehata, Tristan Krap

**Affiliations:** 1 University of Amsterdam, Spui 21, 1012 WX Amsterdam, The Netherlands; 2 Vrije Universiteit Amsterdam, De Boelelaan1105, 1081 HV Amsterdam, The Netherlands; 3 Department of Medical Biology, Section Anatomy & Biomedical Engineering and Physics, Amsterdam Medical Centre, Location Academic Medical Centre, Meibergdreef 9, 1105 AZ Amsterdam, The Netherlands; 4 Maastricht University, Minderbroedersweg 4-6, 6211 LK Maastricht, The Netherlands

**Keywords:** Thermal alteration, Bone, Heat, Cremation, Heat-induced changes

## Abstract

When bone is exposed to thermal stress, the chemical composition changes. This affects bone tissue regeneration after surgery, and these changes can also aid in reconstructing ante-, peri-, and post-mortem events in forensic investigations and past activities on cremation practices in archaeology. However, to date, no complete overview exists on the chemical composition of both fresh and thermally altered bone. Therefore, we aimed (i) to present the chemical composition of fresh bone and (ii) to present an overview of heat-induced chemical changes in bone under both reducing and oxidizing conditions. From the overview, it became clear that some chemical changes occur at a consistent temperature, independent of exposure duration, meaning there is a temperature threshold. However, the occurrence of other chemical changes appeared to be more inter-experimentally variable, and therefore, it is recommended to further investigate these changes.

**Supplementary Information:**

The online version contains supplementary material available at 10.1007/s00414-024-03160-z.

## Introduction

Bone can be exposed to excessive heat under different circumstances, ranging from medical methods that apply heat locally to the bone to funerary practices resulting in complete cremation of a body. An example of a medical method by which heat is applied to bone is the usage of laser scalpels, which can result in local exposure to heat up to 600 °C [1–3]. According to the World Health Organization (WHO), every year more than 300,000 people directly die due to fires, of which approximately 50 in the Netherlands [4, 5]. These fires can be caused by traffic accidents, explosions, bombings, bush fires, and mass disasters, as well as complex suicides and homicides [6, 7]. In these cases, temperatures can go over 1000 °C, depending mostly on the fuel and oxygen availability [8–10]. After such fires (fragmented), thermally altered human skeletal remains (cremains) can be found, which hold evidentiary value for forensic or identification purposes, such as for reconstruction of ante-mortem and post-mortem events [11]. This is of major importance judicially as well as to aid in closure for the relatives of the deceased. The study of human and non-human heated skeletal remains also holds value for archaeological science to better understand historical and cultural practices, such as funerary obsequies or sacrificing [12–14]. In case of deaths caused by fires, frequently, the use of the — by the International Committee of the Red Cross (ICRC) and Interpol named — ‘primary identifiers’ short tandem repeat (STR) DNA analysis, ridgeology, and to a certain extent the analysis of unique medical identifiers, as well as ‘secondary identifiers’, such as tattoos and clothing, are impaired by the fire as well as non-biological forensic traces [7, 15–21]: the only sources for forensic or identification purposes are in some instances solely the bones and teeth. However, the extraction of biological features from cremated remains (cremains), like traumas and pathologies, can be compromised, since this assessment is based on the metrics and morphology of the bone material, while heat can cause morphological alterations, such as colour, size, warping, and fragmentation [6, 22–30]. Therefore, it is of importance to review the heat induced alterations of bone, since these changes can be valuable for medical, forensic, and archaeologic casework, and identification purposes.

Previous research has shown several forensic and archaeological applications of these heat-induced (HI) changes, i.e. the assessment of exposure time and temperature, the heating phases and their morphology, and the chemical composition-dependent photoluminescence of cremains [27, 28, 31–34]. The (change in) chemical composition of bone could be used for estimating the exposure temperature of bones and thus the position(s) of the body during the fire exposure, which could be helpful in the reconstruction of ante-mortem, peri-mortem, and post-mortem events [35]. Also, by accurately estimating the exposure temperature, a well-considered decision on sampling for DNA- and isotope-analysis can be made for identification [36]. However, although extensive research has been performed on the chemical composition of fresh and thermally altered bone, by means of different analytical set-ups, up till now no complete overview of the chemical composition over temperature exists [28, 29, 31–33]. Therefore, the main aim of this literature study is to provide an overview of the chemical composition of fresh and thermally altered bone. In order to do so, first the chemical composition of fresh bone was reviewed, whereupon the heating of bone was discussed. Subsequently, the HI chemical changes were reviewed. This was done by a broad literature search on both Google Scholar and Scopus database, primarily using keyword search. In addition, the references and citations of the selected literature were checked to find additional relevant literature. Given the small number of studies on the chemical composition of fresh and thermally altered bone, no publication date filter was applied. Included were original papers, books and reviews (*see also: Electronic **Supplementary Material*).

## The structure and composition of human bone

### The structure of human bone

Bone tissue has several functions: giving support to the entire body, the storage of minerals and lipids, the production of blood, the protection of soft tissue and organs, and leverage [37]. In order to review the chemical composition of thermally altered bones, it is important to first review the initial structure and chemical composition of fresh bones.

Bone consists of two types of structures: approximately 20 wt% cancellous, spongy, or trabecular bone and 80 wt% cortical or compact bone [38]. Both structure types have the same chemical composition. The bone consists of four cell types, namely osteoblasts, osteocytes osteoclasts, and osteoprogenitor [37]. Two types of canals can be found in the bone: Haversian and Volkmann’s canals. On the inside of both canals the endosteum, a thin vascular membrane, can be found, which is similar to the membrane on the outer surface of bone, the periosteum. In this literature overview, the bone matrix is defined as every element that can be found between the periosteum and the endosteum, meaning that the tissue types that can be found in these canals are not considered as part of the bone matrix. However, since these are not removed in the cremation experiments of the literature this overview is based on, it is important to consider them as well. Through both bone structure types, blood — and thus nutrients — are transported. In cortical bone, this transportation is regulated by vessels, located in the Haversian and Volkmann’s canals. In cancellous bone, trabeculae have formed an open network, wherein nutrients are transported via diffusion, since venules and capillaries are not present here [11, 37]. Aside from blood, also, a semi-solid tissue can be found, namely bone marrow. Two types of bone marrow can be distinguished in the bone: red and yellow bone marrow. The former functions as producer of erythrocytes, lymphocytes, and thrombocytes, whereas the latter mainly functions as storage for adipose tissue [37].

### The chemical composition of human bone

**Fig. 1:**
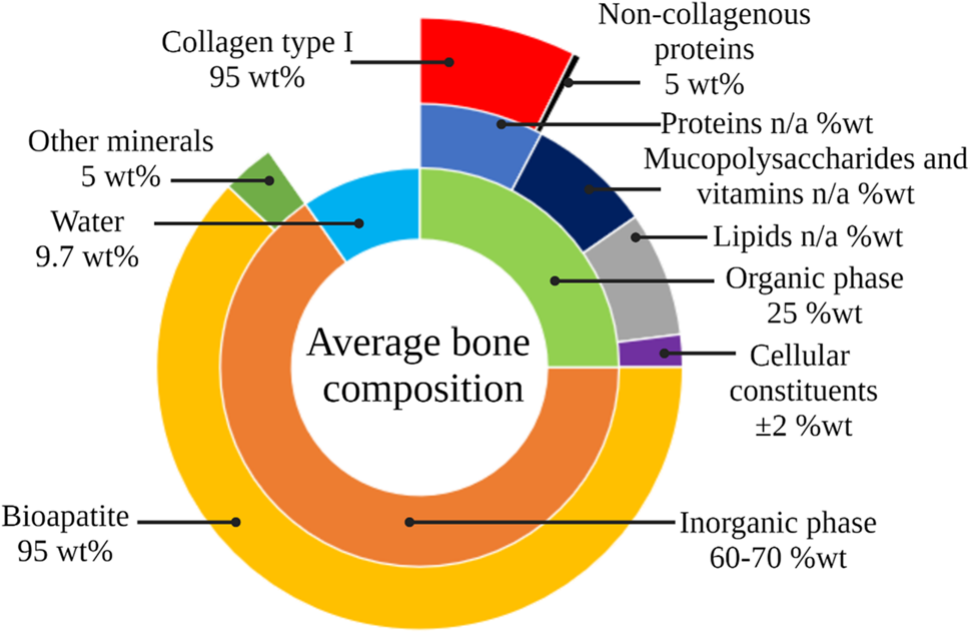
A multi-layered pie chart of the average human bone composition (figure is made in Microsoft Excel)

A general consensus exists on the heterogenous material bone consists of the bone matrix consists of approximately 25 wt% organic constituents, 60 wt% inorganic components (70 wt% for dry bones), and 9.7 wt% water (Fig. 1) [39–44]. Since this overview focusses on the chemical HI changes within the bone, the chemical definition of organic compounds will be utilized: chemical compounds that contain carbon-hydrogen or carbon–carbon bonds.

#### The organic phase

The most abundant organic component in the bone is collagen type I [37, 39]. The fibrils formed from triple helices of collagen are mineralized to a certain degree, determining the rigidity of the tissue [37]. For collagen in the bone, this mineral is mostly bioapatite (BAp), which will be further discussed in the following paragraph. Additionally, so-called non-collagenous proteins (NCPs) can be found in bones, such as albumin and alpha-2-HS-glycoprotein, [37, 38]. Together with lipids, proteins make up 92 wt% of the organic phase of the bones (23 wt% of the bone matrix). Mucopolysaccharides, vitamins (vitamin A, B12, C, D_3_, and K), and a variety of hormones can also be found in the organic phase as well as cellular constituents (2 wt%) [38, 39, 41, 42].

Inside bones, (nutrient) arteries and adipose tissue can be found, and since these are not removed in the heating experiments of the literature this overview is based on, it is important to consider their composition as well. The blood vessels and the red bone marrow — where erythrocytes (± 45%, of which ± 15% the iron-containing protein hemoglobulin), leukocytes (± < 1%), and thrombocytes (± < 1%) are formed from stem cells — consist of water, with approximately 51% of the blood the main component, the above-mentioned cell types (± 45%), and plasma proteins (± 4%), such as albumins, fibrinogens, and globulins [37]. Moreover, electrolytes (± 1%), such as sodium (Na^+^), chloride (Cl^−^), magnesium (Mg^2+^), potassium (K^+^), and carbonate (CO_3_^2−^), can be found in the plasma [30]. Lastly, adipose tissue can be found in the yellow bone marrow. The adipose tissue mainly consists of adipocytes, which are cells largely filled with lipids, and additionally consists of the stromal vascular fraction (SVF) and a variety of immune cells (e.g., adipose tissue macrophages) [37].

#### The inorganic phase

**Fig. 2:**
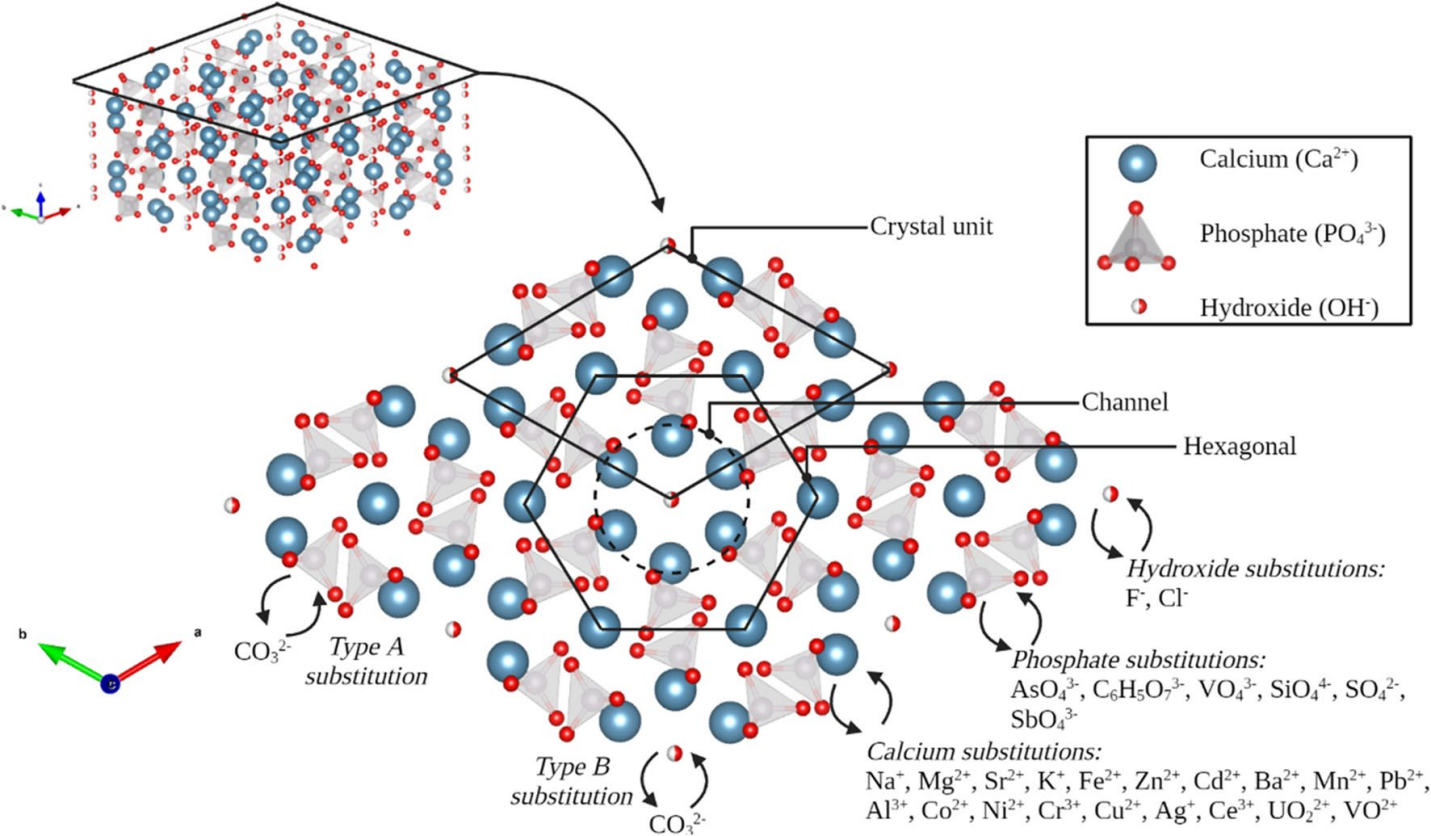
A chemical representation of the hydroxylapatite mineral (Ca_10_(PO_4_)_6_(OH)) and the possible substitutions in the P6_3_/m space group (figure is made with VESTA software and Microsoft PowerPoint [45])

Collagen in the bones is mostly mineralized with BAp, which is the most abundant mineral in the bones [37, 46, 47]. BAp is a salt that consists of calcium (Ca^2+^) and phosphate (PO_4_^3−^) groups, forming a crystal lattice that belongs to hexagonal space group P6_3_/m, which is an ion orientation that is characterized by a six-fold *c*-axis perpendicular to three equivalent *a*-axes at angles of 120° to each other [46, 48, 49]. However, monoclinic space group P2_1_/b is also reported for BAp in literature, which is an ion orientation characterized by a twofold *c*-axis perpendicular to one *a*-axis [46, 48, 50]. As can be observed in Fig. 2, ion channels are present in the salt crystals, which can be occupied by ions. In literature, BAp is often referred to as “hydroxylapatite”, since the general belief was that these anions were mostly hydroxides (OH^−^) and because of their similar spacing symmetry [45]. However, since there is growing evidence for the lack of hydroxide in BAp, in agreement with Wopenka and Pasteris the mineral will be referred to as bioapatite or bone apatite and not hydroxylapatite (HAp) (Ca_5_(PO_4_)_3_OH, which is most often written as idealized unit cell formula Ca_10_(PO_4_)_6_(OH)_2_) [46]. The formula for BAp that is considered the most accurate is a carbonate-substituted HAp: Ca_10_(PO_4_)_6 − *x*_(OH)_2 − *y*_(CO_3_^2−^)_*x* + *y*_ [39]. As can be derived from this formula, carbonate (CO_3_^2−^) can substitute in two manners: type A substitution of the hydroxide groups (OH^−^) and type B substitution of the phosphate groups (PO_4_^3−^), of which the latter is much more common [39, 46, 51–54]. Due to the geometric difference (i.e., the different shape and orientation of hydroxide and carbonate) between the substituted moieties, the crystal lattice is distorted. This results in lower crystallinity and thus small-sized crystals [39, 46, 54, 55].

Factors as diet, place of residence, and metabolism influence the BAp lattice, especially calcium, phosphate, and hydroxide can be substituted by trace elements. An extensive overview of substitutions, found in literature, is depicted in Table 1.

**Table 1:** An overview of calcium, hydroxide, and phosphate substitutions in bioapatite that are found in literature. Per ion the class in the periodic table, the subject in which the trace element is found, and the detection/observation method are indicated. When an ion is indicated to be present without indication of an experimental method, in the second column, ‘Review’ is notated. When an ion contains oxygen or hydrogen, the ion is classified in the class of the other element

Class	Ion	Review or detected/observed with	Substitution type	Subject	Ref
*Alkali metals*	Sodium (Na^+^)	Review	Calcium Calcium Calcium	Fresh human bone	[39]
		Review		Fresh bone and tooth	[46, 56, 57]
		ICP-MS		Fresh human bone	[63]
	Potassium (K^+^)	Review	Calcium Calcium Calcium Calcium	Fresh human bone	[39]
		Review		Fresh bone and tooth	[46, 56]
		XRD		Cremated human bone	[57]
		ICP-MS		Fresh human bone	[63]
	Rubidium (Rb)	ICP-MS	n/a	Fresh human bone	[63]
*Alkaline earth metals*	Magnesium (Mg^2+^)	Review	Calcium Calcium Calcium Calcium	Fresh human bone	[39]
		Review		Fresh bone and tooth	[46, 56, 57]
		ICP-AES		Fresh human bone	[58]
		ICP-MS		Fresh human bone	[63]
	Strontium (Sr^2+^)	Review	Calcium Calcium Calcium Calcium Calcium Calcium Calcium	Fresh human bone	[39]
		Review		Fresh bone and tooth	[46, 56]
		XRD		Cremated human bone	[57]
		Review		Fresh human bone	[59]
		Spectrographic analysis		Fresh human bone	[61]
		Micro-XRF		Fresh human bone	[62]
		ICP-MS		Fresh human bone	[63]
	Barium (Ba^2+^)	Review	Calcium Calcium	Fresh bone and tooth	[57]
		ICP-MS		Fresh human bone	[63]
*Metalloids*	Orthosilicate (SiO_4_^4−^)	Review	Phosphate Phosphate	Fresh bone and tooth	[46, 57]
		Spectrographic analysis		Fresh human bone	[61]
	Arsenate (AsO_4_^3−^)	Review	Phosphate	Fresh bone and tooth	[46, 57]
	Antimonate (SbO_4_^3−^)	Review	Phosphate	Fresh bone and tooth	[46, 57]
*Non-metals*	Citrate (C_6_H_5_O_7_^3−^)	Review	Phosphate	Fresh bone and tooth	[46]
	Sulphate (SO_4_^2−^)	Review	Phosphate	Fresh bone and tooth	[46, 57]
*Halogens*	Fluorine (F^−^)	Review	Hydroxide Hydroxide	Fresh human bone	[39]
		Review		Fresh bone and tooth	[46, 56, 57]
	Chlorine (Cl^−^)	Review	Hydroxide Hydroxide	Fresh human bone	[39]
		Review		Fresh bone and tooth	[46, 56, 57]
*Transition metals*	Scandium (Sc)	ICP-MS	n/a	Fresh human bone	[63]
	Titanium (Ti)	ICP-MS	n/a	Fresh human bone	[63]
	Vanadyl (VO^2+^)	ICP-MS	Calcium Calcium	Fresh and archaeological deer bone	[56]
		ICP-MS		Fresh human bone	[63]
	Vanadate (VO_4_^3−^)	Review	Phosphate Phosphate	Fresh bone and tooth	[46, 57]
		Spectrographic analysis		Fresh human bone	[61]
	Chromium (Cr^3+^)	ICP-AES	Calcium Calcium	Fresh human bone	[58]
		ICP-MS		Fresh human bone	[63]
	Manganese (Mn^2+^)	Review	Calcium Calcium Calcium Calcium	Fresh bone and tooth	[57]
		ICP-AES		Fresh human bone	[58]
		Spectrographic analysis		Fresh human bone	[61]
		ICP-MS		Fresh human bone	[63]
	Iron (Fe^2+^)	Review	Calcium Calcium Calcium Calcium Calcium	Fresh bone and tooth	[46]
		XRD		Cremated human bone	[57]
		ICP-AES		Fresh human bone	[58]
		Spectrographic analysis		Fresh human bone	[61]
		ICP-MS		Fresh human bone	[63]
	Cobalt (Co^2+^)	ICP-AES	Calcium Calcium	Fresh human bone	[63]
		ICP-MS		Fresh human bone	[63]
	Nickel (Ni^2+^)	ICP-AES	Calcium	Fresh human bone	[58]
	Copper (Cu^2+^)	ICP-AES	Calcium Calcium Calcium Calcium	Fresh human bone	[58]
		Review		Fresh human bone	[59]
		Spectrographic analysis		Fresh human bone	[61]
		ICP-MS		Fresh human bone	[63]
	Zinc (Zn^2+^)	Review	Calcium Calcium Calcium Calcium Calcium Calcium Calcium	Fresh bone and tooth	[46]
		XRD		Cremated human bone	[57]
		ICP-AES		Fresh human bone	[58]
		Review		Fresh human bone	[59]
		Spectrographic analysis		Fresh human bone	[61]
		Micro-XRF		Fresh human bone	[62]
		ICP-MS		Fresh human bone	[63]
*Transition metals*	Molybdenum (Mo)	ICP-MS	n/a	Fresh human bone	[63]
	Silver (Ag^+^)	ICP-AES	Calcium Calcium	Fresh human bone	[58]
		Review		Fresh human bone	[59]
	Cadmium (Cd^2+^)	Review	Calcium Calcium Calcium	Fresh bone and tooth	[57]
		ICP-AES		Fresh human bone	[58]
		ICP-MS		Fresh human bone	[63]
*Post-transition metals*	Aluminium (Al^3+^)	Review	Calcium Calcium Calcium Calcium	Fresh bone and tooth	[56]
		ICP-AES		Fresh human bone	[58]
		Spectrographic analysis		Fresh human bone	[61]
		ICP-MS		Fresh human bone	[63]
	Tin (Sn)	Spectrographic analysis	n/a	Fresh human bone	[61]
	Lead (Pb^2+^)	Review	Calcium Calcium Calcium Calcium Calcium	Fresh bone and tooth	[57]
		ICP-AES		Fresh human bone	[58]
		Spectrographic analysis		Fresh human bone	[61]
		Micro-XRF		Fresh human bone	[62]
		ICP-MS		Fresh human bone	[63]
*Lanthanides*	Lanthanium (La)	ICP-MS	n/a	Fresh human bone	[63]
	Cerium (Ce^3+^)	Review	Calcium Calcium	Fresh human bone	[59]
		ICP-MS		Fresh human bone	[63]
	Praseodymium (Pr)	ICP-MS	n/a	Fresh human bone	[63]
	Neodymium (Nd)	ICP-MS	n/a	Fresh human bone	[63]
	Samarium (Sm)	ICP-MS	n/a	Fresh human bone	[63]
	Europium (Eu)	ICP-MS	n/a	Fresh human bone	[63]
	Gadolinium (Gd)	ICP-MS	n/a	Fresh human bone	[63]
	Terbium (Tb)	ICP-MS	n/a	Fresh human bone	[63]
	Dysprosium (Dy)	ICP-MS	n/a	Fresh human bone	[63]
	Holmium (Ho)	ICP-MS	n/a	Fresh human bone	[63]
	Erbium (Er)	ICP-MS	n/a	Fresh human bone	[63]
	Thulium (Tm)	ICP-MS	n/a	Fresh human bone	[63]
	Ytterbium (Yb)	ICP-MS	n/a	Fresh human bone	[63]
	Lutetium (Lu)	ICP-MS	n/a	Fresh human bone	[63]
*Actinides*	Thorium (Th)	ICP-MS	n/a	Fresh human bone	[63]
	Uranyl (UO_2_^2+^)	ICP-MS	Calcium Calcium Calcium	Fresh and archaeological deer bone	[56]
		ICP-MS		Archived human bone	[60]
		ICP-MS		Fresh human bone	[63]

Aside from BAp, minor other calcium phosphates can be found in the bone matrix, namely brushite (CaHPO_4_.2H_2_O), octacalcium phosphate (Ca_8_H_2_(PO_4_)_6_ .5H_2_O), tricalcium phosphate (β-TCP, β-Ca_3_(PO_4_)_2_), calcium pyrophosphate dihydrate (Ca_2_P_2_O_7_), and amorphous calcium phosphates.

Moreover, magnesium phosphates struvite (MgNH_4_PO_4_ ·6H_2_O), newberyite (MgHPO_4_ ·3H_2_O), amorphous calcium magnesium pyrophosphate, and calcium magnesium phosphate have been identified in the human bone matrix [64, 65].

#### The crystal structure of bioapatite

As can be derived from the chemical formula of hydroxyapatite, Ca_10_(PO_4_)_6_(OH)_2_, the Ca/P ratio that should be found in natural HAp is 1.67. In fact, this ratio is higher (1.67–2.0), due to the non-stoichiometry and substitutions that can be found in BAp [46]. This results in the fact that the hexagonal phase (P6_3_/m) is favourable over the monoclinic form (P2_1_/b), which is easily destabilized by substitutions [66]. Although the crystal size is dependent on substitutions, which is person dependent, the average crystal size in unaltered bone is 50 × 25 × 2–4 nm [66].

## The heating mechanisms of human bones

Several heat transfer modes exist, including convection, conduction, and thermal radiation. As a result of the transfer of heat, in bone heating experiments, a distinction should be made between two types of molecular heat changing methods, both are in effect during burning of a human body, namely, combustion and pyrolysis (thermolysis).

For combustion, three ingredients are required: fuel (the reductant), an oxidant (often atmospheric oxygen), and heat. During combustion, the reductant reacts with the oxidant to form more energetically favourable combustion products (negative enthalpy; second law of thermodynamics). However, to be able to react, thermal energy is necessary to overcome the reaction’s activation energy (a thermal threshold). In the case of combustion, the molecules’ thermal energy is influenced by the environmental thermal energy: the higher the environmental thermal energy, the faster the movements of the molecules, the higher the chance that the reductant and the oxidant undergo a successful (i.e., with sufficient energy to overcome the activation energy and with the proper orientation) collision (collision theory).

Pyrolysis takes place when a material is heated under reducing conditions (absence of dioxygen). Due to the absence of reactive dioxygen, heat cannot be used to overcome an activation energy. Hence, heat in the form of kinetic energy is transferred to the atoms. When the decomposition temperature of the compound is reached, the kinetic energy of the atoms in a substance is too high to maintain the binding energy, causing the bonds to break, forming smaller molecules and/or free atoms. Since the two heating mechanisms are completely different, the products that are formed during a combustion will differ from the products that are formed from pyrolysis.

Aside from the temperature, also insulation (i.e., clothing or subcutaneous fat), critical mass, and surface area of the bones, as well as the exposure time influence the degree of pyrolysis or combustion, the latter being the most influential for bone cremation experiments [67]. When the exposure time increases, while the temperature is stable, more thermal energy is transferred to the substance’s atoms, resulting in an increase of the atoms’ kinetic energy and thus higher pyrolysis kinetics, which will continue until only free elements are left. For combustion, the chance of a successful collision increases, when the exposure time is increased, because the molecules move faster for a longer duration. Therefore, for comparison of experiments, it would be most useful to express the experimental conditions in for example the accumulated thermal unit (ATU), which is the exposure duration in days multiplied with temperature in degrees Celsius (°C). Unfortunately, in literature, temperatures are most often coupled to chemical changes. Moreover, one should differentiate between the two heating mechanisms pyrolysis and combustion used in experiments (both occur when tissues are heated of a human body).

## Heat-induced chemical changes in human bone

During exposure to high temperatures and fire, the chemical composition and structural properties of human bone are altered.

**Fig. 3:**
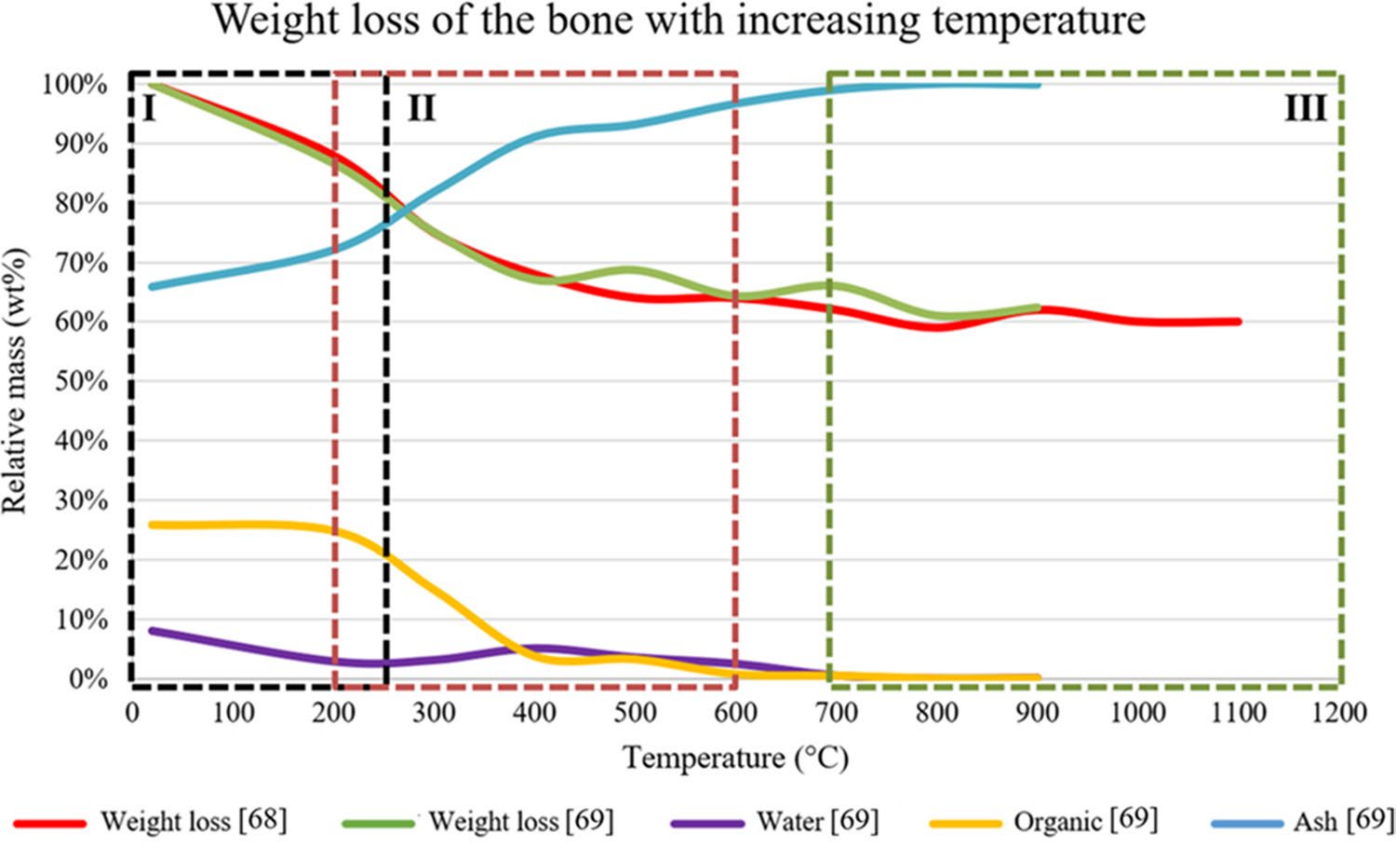
A graphical representation of the weight loss of bone with increasing temperature, as described by Imaizumi et al. [68] and Van Hoensel et al. (2019). The three stages are indicated by boxes with a corresponding roman number

Imaizumi et al. as well as Van Hoensel et al. (2019) measured the mass loss of the bones during the (controlled) heating process (Fig. 3) [68, 69]. This will be the *terminus a quo* to describe the HI changes, since three stages with chemical changes can be clearly observed: (i) the loss of water below 250 °C (I), (ii) a decline in organic content between 200 and 600 °C (II), and (iii) changes of the bone mineral above 700 °C (III).

### Water

During the first stage, below 250 °C, the main alteration in the bone is the loss of water [69, 70]. According to Etok et al. [70] and Van Hoensel et al. (2019), first adsorbed water will evaporate from the bone, up to 100 °C, where after the structural bound water from proteins and mineral surfaces is lost [69–71]. Both measured this with thermogravimetric analysis (TGA) and Fourier transform infrared spectroscopy (FTIR). However, the appositeness of methods, such as TGA, can be debated, since the bone sample is ramped (i.e., a repetition of increasing the temperature and measuring the chemical composition). Although the effect of ramping on the HI changes is still unknown, it is expected this does affect the temperature a HI change occurs, given that the exposure expressed in ATUs is much higher for ramped bone than for bone that is heated a single time. As can be seen in Fig. 3, most water has been evaporated at 250 °C, but in the second stage, around 400 °C, the concentration of water increases slightly. According to Shafizadeh et al. [72], water loss at higher temperatures is due to the loss of structural water from the organic layer and as a result of thermal degradation [72]. An increase can be seen around 400 °C, which could be explained by the major loss of organic compounds between 300 and 400 °C, where water is formed as reaction product of combustion. It is suggested that only a slight increase can be observed around 400 °C, because the water that is formed, evaporates immediately at these temperatures. As a result of the degradation into even smaller organic compounds, Van Hoensel et al. (2019) measured the formation and immediate evaporation of water with FTIR up to 700 °C [69]. Similar observations were done by Reidsma et al. [73] under reducing conditions [73].

### Proteins

In the second stage, the bone loses most of its weight, which is mainly due to the loss of organic components (Fig. 3). Aside from FTIR, Van Hoensel et al. (2019) performed pyrolysis mass spectrometry (pyMS) on the bone and observed minor changes in the organic composition up to 200 °C [69]. Between 200 and 350 °C, a decrease of the most abundant protein, collagen, is observed. The degradation of collagen is accompanied by the introduction of new compounds, namely alkylated phenols, alkylated benzenes, condensed aromatic compounds, and N-containing heterocyclic compounds between 300 and 340 °C [63, 68]. Reidsma et al. [73] found similar temperatures under reducing conditions with direct temperature-resolved mass spectrometry (DTMS) [73]. From 350 °C onwards, smaller thermally degraded compounds were observed, such as benzene, until these aromatic compounds were completely oxidized and not observable anymore with FTIR and TGA at 600 °C (Reidsma et al. [73]: 900 °C with DTMS under reducing conditions) [73]. Van Hoensel et al. (2019) have made a distinction between combusted bone (i.e., heated in the presence of oxygen) and charred bone (i.e., heated under reducing conditions). While in the former the organic phase is mostly (93.5%) combusted at 350 °C, the charred bone has lost a similar percentage of organic phase at 600–700 °C [69]. Moreover, above 600 °C or 700 °C, cyanamide (CH_2_N_2_) was detected with FTIR and inelastic neutron scattering (INS), only for the charred bone [73, 75]. The presence of organic phase at higher temperature is in agreement with the observation of Reidsma et al. [73] to find organic compounds at 900 °C, since these experiments were performed under reducing conditions [73]. Similar to the aromatic compounds, the concentration of formed nitrogen containing (inorganic) compounds, such as prussic acid (HCN) and acetonitrile (ACN), is strongly reduced above 350 °C. A visibly observable, and measurable, effect of the thermal degradation of collagen is the HI-change in colour of the bone, from ivory white fresh bone to black when carbonized and ashy grey to calcined white after exposure to temperatures above 500 °C [68, 76–78].

According to Reidsma et al. [73], no proteins were detected anymore under reducing conditions at temperatures of more than 370 °C, while Correia [79] reported the presence of collagen at temperatures up to 800 °C after microscopic observations, Castillo et al. [29] up to 600 °C, and Marques et al. [80] reported a complete combustion of proteins between 700 and 900 °C with FTIR-attenuated total reflectance (ATR) and INS [29, 79, 80]. The inconsistency between the authors here could be explained by the definition of ‘a complete combustion of proteins’. On the one hand, this could include the combustion of their organic degradation products. On the other hand, this could solely be the combustion until no protein is left anymore. The difference between the results of Reidsma et al. [73] and the other authors could be explained by the availability of oxygen. However, keeping in mind the other HI changes, the temperature is high. The results of Reidsma et al. [73] are considered more reliable than the results of Correia [79] and Castillo et al. [29], since analytical methods are preferred over subjective interpretation of histological features [81, 82]. So, although proteins, such as collagen, have shown to endure high temperatures, it is expected that these will be broken down before the third stage at 700 °C, and will certainly not be present in their original form, neither under oxidizing conditions [47].

### Yellow bone marrow, adipose tissue, and lipids

Consensus exists regarding the thermal degradation of adipose tissue: Van Hoensel et al. (2019) measured the HI change of lipids in the bone: the thermal degradation of lipids is suggested to be completed below 300 °C after pyMS analysis, since no lipid markers were found above this temperature [69]. Braadbaart [74] reported a similar temperature between 340 and 370 °C for the evaporation of lipids (in sunflower seeds) [83]. Reidsma et al. [73] reported the completion of the thermal degradation of lipids at a temperature of 340 °C under reducing conditions after DTMS analysis [73]. So, the lipid content of the human bone is completely degraded in the second stage.

### Hormones and vitamins

Several hormones and vitamins can be found in the bone [37–39, 41, 42]. To the best of our knowledge and based on the literature search, no research has been performed on the thermal degradation of these compounds in bone. Considering the chemical structure of vitamins and hormones, it is supposed that the thermal degradation will be similar to the organic compounds that originated from protein degradation, meaning a complete degradation around 600 °C in the presence of oxygen and 900 °C under reducing conditions [69, 73]. Note, vitamin B12, that can also be found in the bone (mostly in the red blood cells and red bone marrow), is a coordination complex of cobalt (Co^+^) and certain ligands. The cobalt ion will not be thermally degraded and will remain in the bone [37].

### Red bone marrow and blood

As discussed, a major part of the organic phase is thermally degraded between 300 and 400 °C. Although some HI physical conversions, or denaturations, within the blood are described in literature, such as the HI haemolysis at 52 °C and DNA denaturation between approximately 60 and 100 °C, to the best of our knowledge, no literature can be found on HI chemical conversions of specifically blood and/or red bone marrow components [84, 85]. Therefore, it is assumed that the proteins in blood and red bone marrow as well as the other organic components (e.g., cellular constituents) and water in the blood follow the same reaction pathways as respectively the organic components and water within the bone matrix. However, the electrolytes sodium (Na^+^), chloride (Cl^−^), magnesium (Mg^2+^), potassium (K^+^), and carbonate (CO_3_^2−^), that can be found in the plasma, will not be degraded [37]. Moreover, the main component of blood, haemoglobin, is an iron-containing metalloprotein consisting of heme groups that contain four pyrrole molecules and an iron ion (Fe^2+^ or Fe^3+^ for met-haemoglobin) [30]. These cations will still be present in the bone after thermal degradation of the organic phase. So, from the blood and red bone marrow, only some ions will still be present during the calcination stage (Correia [79]: 700–1100 °C).

### Carbon oxides, (calcium) carbonates, and calcium oxides

During the whole second phase, a strong presence of carbon monoxide (CO) and carbon dioxide (CO_2_) was observed, which is between 250 and 500 °C mostly due to the (incomplete) combustion of the, by protein degradation, formed compounds, the proteins themselves, and the lipids (Eq. 1) (between 300 and 500 °C, according to Etok et al. [70]) [39, 69, 70].

1 $$\mathrm a\;{\mathrm C}_{\mathrm x}{\mathrm H}_{\mathrm y}{\mathrm O}_{\mathrm z}+\mathrm b\;{\mathrm O}_2\xrightarrow{\mathrm T}\mathrm c\;{\mathrm{CO}}_2+\mathrm d\;\mathrm{CO}+\mathrm e\;{\mathrm H}_2\mathrm O$$

Equation 1. A chemical representation of a general incomplete combustion of an organic compound into carbon dioxide, carbon monoxide, and water. The coefficients and subscripts are denoted as letters.

As can be seen from the upper equation, and Fig. 3, water is formed during combustion, which is also observed with FTIR (until 700 °C) [69]. However, this will evaporate immediately at these temperatures (see also: 4.1 Water).

According to i.a. Mamede et al.[39], a second fraction (50% according to Etok et al. [70]) of carbon dioxide was released at temperatures of more than 500 °C. Structural carbonate (CO_3_^2−^) loss from the bone matrix (i.e., in BAp) is suggested as source [39, 55, 70, 86]. Reidsma et al. [73] measured a minor carbonate loss under reducing conditions at much lower temperatures, namely between 250 and 340 °C, with FTIR. However, most carbonate loss was measured above 600 °C [73]. Van Hoensel et al. (2019) confirmed the carbonate loss with FTIR and TGA under oxidizing conditions and also observed a release of water up to 700 °C and therefore proposed the following equations:

2 $$\mathrm{CO}_3^{2-}+{\mathrm H}_2\mathrm O\xrightarrow{\mathrm T}{\mathrm{CO}}_2+2\;\mathrm{OH}^-$$

3 $$\mathrm{CO}_3^{2-}+2\;\mathrm{HP}\mathrm O_4^{2-}\xrightarrow{\mathrm T}{\mathrm{CO}}_2+2\;\mathrm{OH}^-+{\mathrm P}_2\mathrm O_7^{4-}$$

Equations 2 and 3. A chemical representation of two possible reaction equations for the conversion of carbonate into carbon dioxide and the rehydroxylation of bioapatite [69].

Equation 2. was considered the most likely, since no evidence was found for the presence of pyrophosphate (P_2_O_7_^4−^) with FTIR [69]. Moreover, Mamede et al. [14] measured an increasing hydroxyl content in bioapatite between 700 and 900 °C with FTIR-ATR [14]. This is further supported by the observation of carbon trioxide (CO_3_) by Van Hoensel et al. [69] and Madupalli et al. [87], of which a first peak is observed at 600 °C with FTIR. It is suggested that the carbonate ions reorganized into carbon trioxide to create space for the formed hydroxyl ions [69, 87]. Under reducing conditions, Reidsma et al. [73] did not found any evidence for Eq. 3 [73].

Amongst others, Marques et al. [80] and Haberko et al. [88]reported again a carbonate loss in the third stage, between 700 and 1100 °C, with the major loss below 1000 °C [79, 80, 88]. This was measured with different methods, such as carbonate precipitation from the bone and FTIR-ATR. At the same time, Piga et al. (2011) observed the appearance of calcium oxide or lime (CaO) at temperatures of 775 °C with powder X-ray diffraction (p-XRD), increasing up to 1000 °C [89]. With general XRD, Van Hoensel et al. (2019) observed the formation of lime above 800 °C, Rogers and Daniels. [90] and Haberko et al. [88] above 700 °C, and others above 900 °C [64, 69, 88, 90, 91]. The discrepancy between temperatures can be explained by the differences in the age of the donors [90]. Best et al. [92] suggest that lime is formed during the formation of β-TCP when the Ca/P-ratio is higher than 1.67 [92]. Piga et al. (2011, 2018) therefore proposed the following chemical reaction:

4 $$2\;{\mathrm{Ca}}_5{(\mathrm{PO}}_4)_3\mathrm{OH}\xrightarrow{\mathrm T}3\;{\mathrm{Ca}}_3{(\mathrm{PO}}_4)_2+\mathrm{CaO}+{\mathrm H}_2\mathrm O$$

Equation 4. A chemical representation of the conversion of HAp into β-TCP, calcium oxide, and water [89, 93].

In the case that water does not evaporate completely in Eq. 4, which is dependent on the speed of cooling after the burning process, also rehydrated calcium hydroxide (Ca(OH)_2_) was found with p-XRD [89].

However, to the best of our knowledge and based on the literature search, in literature, no reaction equation is proposed for carbonate-substituted HAp, while this could take place, since carbonate-substituted HAp is still present when lime is already formed. Moreover, Piga et al. (2011) also found calcite (CaCO_3_) with p-XRD at a temperature of 1050 °C [89]. Also, Rogers and Daniels. [90]measured the release of water as well as carbon dioxide during the formation of lime with XRD, suggesting an additional chemical reaction [90]. Therefore, the following chemical reaction is hereby proposed:

5 $${\mathrm{Ca}}_{10}{(\mathrm{PO}}_4)_6{\mathrm{CO}}_3\xrightarrow{\mathrm T}3\;{\mathrm{Ca}}_3{(\mathrm{PO}}_4)_2+{\mathrm{CaCO}}_3\xrightarrow{\mathrm T}3\;{\mathrm{Ca}}_3{(\mathrm{PO}}_4)_2+\mathrm{CaO}+{\mathrm{CO}}_2$$

Equation 5. A chemical representation of the conversion of carbonate substituted HAp into β-TCP and calcite, which is further degraded into calcium oxide and carbon dioxide.

Under reducing conditions, Reidsma et al. [73] did not observe the formation of lime at high temperatures (900 °C) at all [73]. However, lime could be formed at even higher temperatures under reducing conditions at which no experiments are performed (yet).

Aside from lime, also buchwaldite (NaCaPO_4_) [87], magnesium oxide (MgO) [89], sodium chloride (NaCl) [39], and potassium chloride (KCl) [39] are observed, which could be explained by the presence of substituting ions in the initial BAp lattice and the presence of other minerals in unaltered bones [89].

### Bioapatite and tricalcium phosphate

The HI changes of the most abundant inorganic compound in the human bones can be found mostly in the third stage. The BAp crystals are formed during the mineralization of the collagen fibrils. A general consensus exists that the mineralization process is accelerated at higher temperatures and that the crystal size increases non-linearly with increasing temperature under both reducing and oxidizing conditions [34, 39, 73, 88, 94–96]. Mamede et al. reported the first increase of the crystal size between 300 and 500 °C [39]. Shipman et al. measured a slow increase between room temperature and 525 °C and a steep increase between 770 and 800 °C with XRD [94]. Also, Figueiredo et al. measured an increase from 63 nm at 600 °C to 76 nm at 900 °C to 105 nm at 1200 °C with XRD, which were similar results as obtained by Haberko et al. (2006) [88, 95]. Holden et al. [91] performed extensive scanning electron microscopy (SEM) on the BAp crystal sizes and shapes and also observed an increase in crystal size at higher temperatures for the different crystal shapes observed: for spherical crystals the crystal size increased from 64 nm at 600 °C to 200 nm at 1200 °C and for hexagonal crystals from 300 nm at 800 °C to 1200 nm at 1200 °C [96]. However, one must consider this was performed with a microscopic method. Moreover, the amount and type of substitutions influence the initial crystal size, e.g., the more carbonate substitutions, the smaller the crystals [46]. Therefore, the trend is more informative than the absolute crystal sizes.

**Fig. 4:**
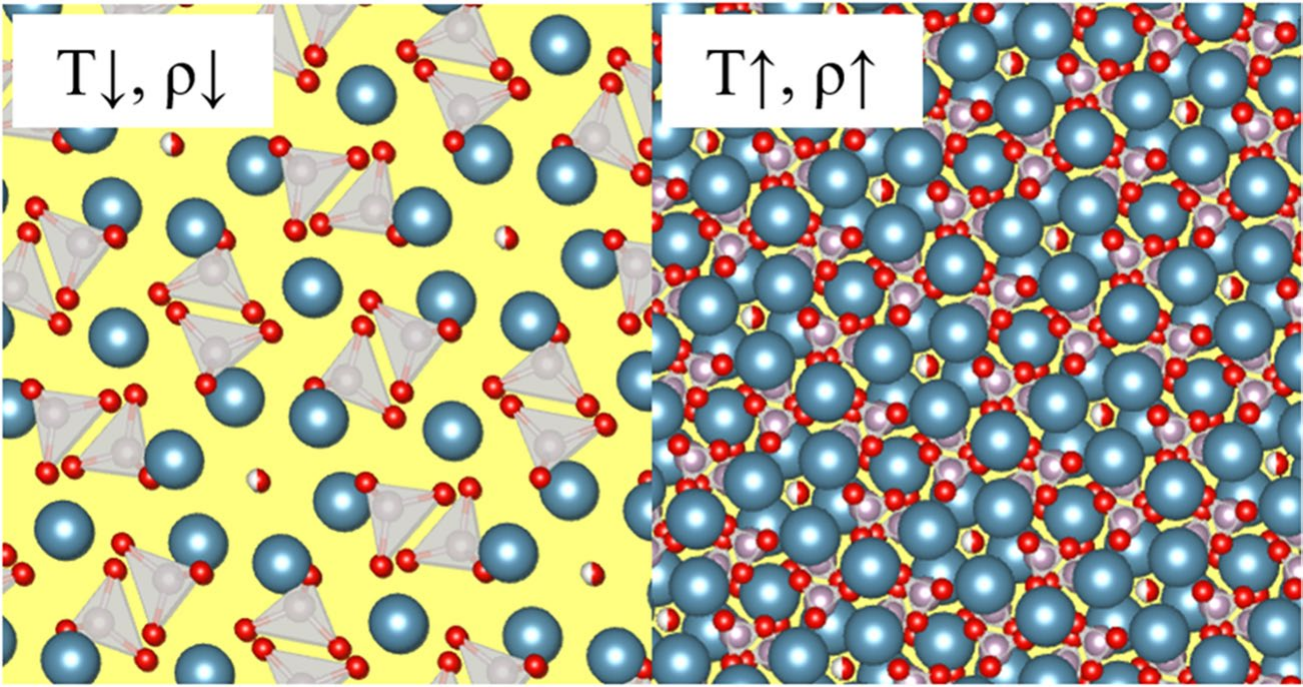
A chemical representation of the hydroxylapatite mineral (Ca_10_(PO_4_)_6_(OH)) with different densities that are caused by exposure to different temperatures. The left figure is the true crystal density at room temperature; the left crystal density is an exaggerated increase of the crystal density at higher temperatures (crystal structures are made with VESTA software [45])

Imaizumi et al. [68] found that the volume and weight loss of the bone are not directly proportional and therefore from 500 °C onwards an increase in density was observed [68]. The density increased slowly between 500 and 700 °C (67 to 74%) and faster between 700 and 1100 °C (74 to 128%) [68]. A similar trend was measured for Vickers microhardness of the bone by Fredericks et al. (2015) [97]. The increase in density and Vickers microhardness could be explained by the BAp crystals growing and thus rearranging slowly between 500 and 700 °C and a steeping growth at higher temperatures (Fig. 4).

**Fig. 5:**
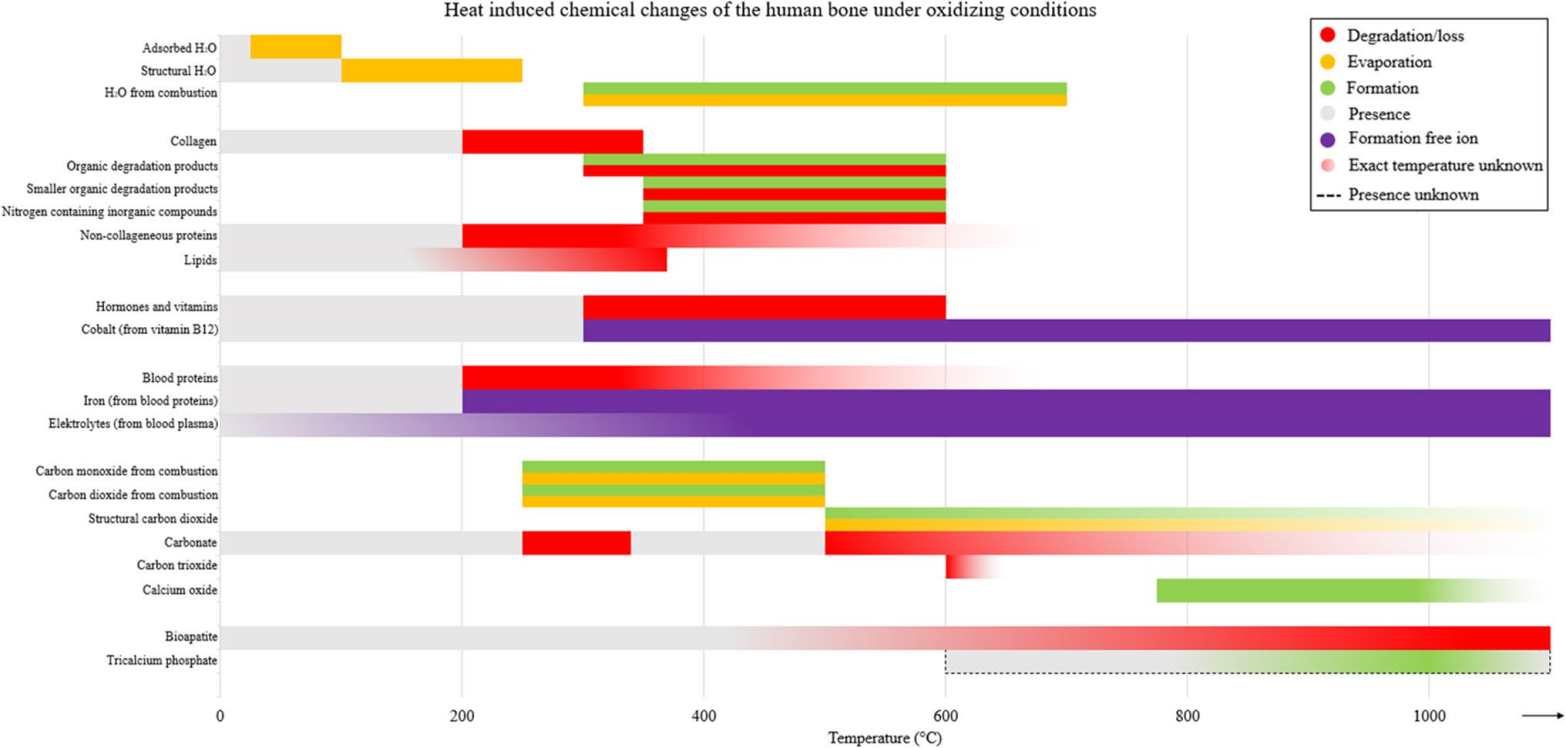
An overview of chemical HI changes of the human bone under oxidizing conditions. With the dotted double arrow line, the calcination phase is visualized (Figure is made in Microsoft Excel)

**Fig. 6:**
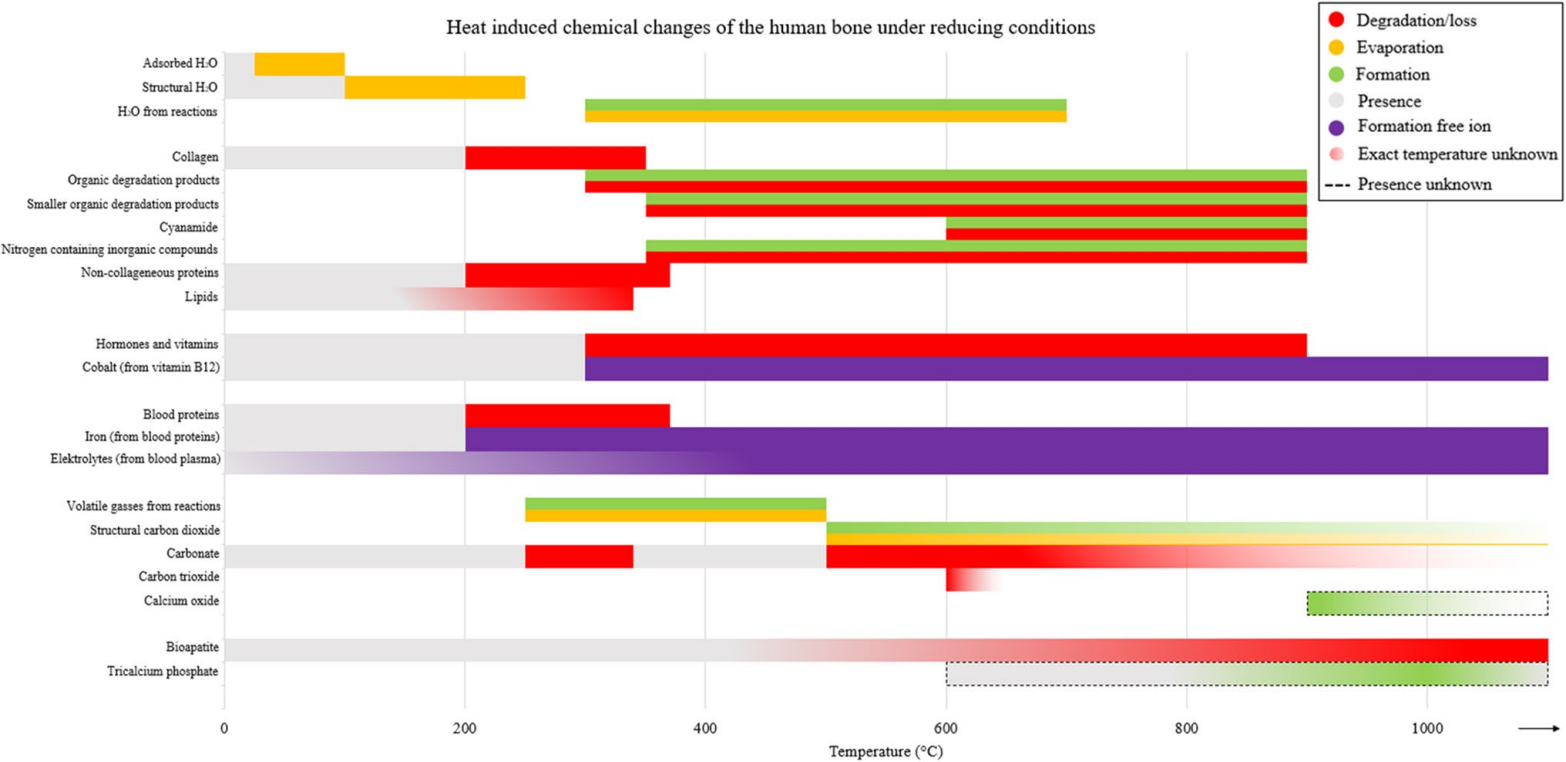
An overview of chemical HI changes of the human bone under reducing conditions. With the dotted double arrow line, the calcination phase is visualized (Figure is made in Microsoft Excel)

As discussed in “Carbon oxides, (calcium) carbonates, and calcium oxides,” at higher temperatures BAp is converted into β-TCP and lime. However, also, the other crystalline polymorph α-TCP is formed [79, 98, 99]. This is assumed to be the cause of the changes in crystal morphology. Similar to the formation temperature of lime, no consensus exists on the formation temperature of β-TCP. Mamede et al. reported a formation temperature of above 1000 °C, while Civjen et al. (1972) and Bonucci and Graziani reported a temperature between 600 and 800 °C after TGA, Rogers and Daniels did not observe the formation of β-TCP between 20 and 1200 °C at all with XRD, and Piga et al. measured β-TCP at temperatures above 1100 °C with XRD [39, 90, 93, 98, 99]. Similarly to Rogers and Daniels. [90], Van Hoensel et al. (2019), and Reidsma et al. [73] did not observe the formation of β-TCP under respectively oxidizing and reducing conditions [69, 73]. The inconsistency here could be due to difficulties in distinguishing β-TCP from BAp with the utilized methods, since both have a similar structure. An overview of the chemical HI changes is provided in Figs. 5 and 6, under respectively oxidizing and reducing conditions.

## Conclusions and future recommendations

The aim was to provide an, to date non-existing, overview of the chemical composition of fresh and thermally altered bone. While no claim can be made that all existing literature, that could be useful to reach the aim, was found, considerable effort was put into providing an as complete overview as possible. This resulted in an extensive overview of heat-induced chemical changes over temperature under both reducing and oxidizing conditions. With this overview more insight is provided into heat-induced changes, which holds value for the use of methods by which heat is applied to bone, to assess the degree of change, or when the degree of heat induced chance is estimated from bone. Furthermore, we proposed a new hypothesis regarding the thermal decomposition of bioapatite into β-TCP and calcite.

There is sufficient evidence in literature to substantiate temperature thresholds that can be applied in practice. Therefore, when chemical analysis is applied, it is valid to draw conclusions from the two most scientifically-grounded HI changes: the presence or absence of organic compounds to distinguish lower from higher exposure temperatures, which is important to make a decision regarding DNA-sampling, and the presence or absence of lime to distinguish the higher exposure temperatures ranges when dealing with calcined human remains. However, the results of this review also show that a lot of other aspects of the scientific basis have not sufficiently been demonstrated yet with chemical analyses. The application of chemical analysis for HI changes, for forensics and archaeological practice, should only be applied with sufficient empirical proof and understanding of the HI-change.

Due to discrepancies in literature, on the heat-induced changes of human bones, it remains difficult to draw conclusions in case work. To understand HI-changes, an interdisciplinary approach, as was here applied, is required. More frequent and intensive cooperation between disciplines is, therefore, recommended. It is also recommended to perform non-controlled experiments with for example whole human bodies to obtain data of chemical heat-induced changes of the bone. Furthermore, it is recommended for future studies to investigate the use of accumulated thermal units, which can make it less difficult to compare results and may in the end result in a multidimensional overview by which environmental conditions could be inferred from the chemical changes (or even other heat-induced changes, such as spectral photoluminescence data). This can be helpful in further developing a method with which ante-mortem, peri-mortem, and post-mortem events can be reconstructed, which could be useful for both forensics and archaeology.

### Supplementary Information

Below is the link to the electronic supplementary material.

Supplementary file1 (DOCX 22 KB)
