# Supplementary material for: An overview of the heat-induced changes of the chemical composition of bone from fresh to calcined
Source: Int J Legal Med. 2024 Jan 25;138(3):1039–53. doi: 10.1007/s00414-024-03160-z (PMC11004044; doi:10.1007/s00414-024-03160-z)
Supplement: Supplementary file 1 — Supplementary file1 (DOCX 22 KB) [file 414_2024_3160_MOESM1_ESM.docx]

**Heat-induced changes of the chemical composition of bone from fresh to calcined**

**Thomas P. Shehata^a,b^, Tristan Krap^c,d^**

- 1. *University of Amsterdam, Spui 21, 1012 WX Amsterdam, The Netherlands*

1. *Vrije Universiteit Amsterdam, De Boelelaan 1105, 1081 HV Amsterdam, The Netherlands*
2. *Department of Medical Biology, Section Anatomy & Biomedical Engineering and Physics, Amsterdam Medical Centre, Location Academic Medical Centre, Meibergdreef 9, 1105 AZ Amsterdam, The Netherlands*
3. *Maastricht University, Minderbroedersweg 4-6, 6211 LK Maastricht, The Netherlands*

**Corresponding author**

Tristan Krap

**Email:** [*t.krap@maastrichtuniversity.nl*](mailto:t.krap@maastrichtuniversity.nl)

**Postal address:** *Minderbroedersweg 4-6, 6211 LK Maastricht, The Netherlands*

**Electronic Supplementary Material – Search Strategy**

**ESM1. Inclusion criteria**

The literature search was conducted between September 6^th^, 2022 and March 1^st^, 2023. Publications from multiple disciplines were included: (analytical) chemistry, (forensic) anthropology, forensic science, geology, (forensic) archaeology, (forensic and legal) medicine, biochemistry, radiology, crystallography or a combination of these. Publications were included if they fell into one or more of these three categories:

1. They contained information on the chemical composition of fresh bone or materials similar to fresh bone.
2. They contained information on the heat-induced chemical changes in bone or materials similar to bone.
3. They contained information on the legal framework the study of heat-induced chemical changes in bone could be valuable for.

In addition to these requirements, only English publications were included. Given the small number of studies on the chemical composition of fresh and thermally altered bone, no publication date filter was applied.

**ESM2. Initial literature**

Some literature was provided as initial literature, namely [19, 28, 40, 50, 59]. These were provided, since these were cited by or published by one of the authors of this publication in earlier publications. These were also included if they met the inclusion criteria, but do not fall under any of the search methods below.

**ESM3. Keyword Search Google Scholar and Scopus Database**

Google Scholar and Scopus Database were selected as search engines, since these databases offer advanced search functionality and also contain a wide variety of publications. Several keywords from the initial literature were used as keywords to start the literature search, namely: ‘burned bone’, ‘heat-induced change’, ‘cremains’, and ‘cremation’. Since these keywords were insufficiently specific on their one, an additional keyword was often added to these keywords, as depicted in **Table 1.** under ‘Keyword 2’. Moreover, when searching for specific information, (the combination of) keyword 1 and 2 was/were supplemented by ‘Keyword 3’.

**Table 1.:** (Combination of) keywords used during the literature search:

| **Keyword 1** | **Keyword 2** | **Keyword 3** |
| --- | --- | --- |
| Burned bone | Human bone | Bioapatite |
| Heat-induced change | Chemical composition | Hydroxyapatite |
| Cremains | Cremated remains | Legal framework |
| Cremation | Heat | DNA-analysis |
|  | Heating | Histology |
|  | Reducing conditions | Organic phase |
|  | Oxidizing conditions | Inorganic phase |
|  | Chemical alteration | Crystal lattice |
|  |  | Substitutions |
|  |  | Blood |
|  |  | Bone marrow |
|  |  | Lipids |
|  |  | Carbonate |
|  |  | Tricalcium phosphate |
|  |  | Collagen |

**ESM4. Citation Search**

Both the initial literature and the publications found with the keyword search were used to search for other relevant publications, making use of the references/citations. The purpose of this second stage was to find relevant literature that did not meet the keyword search criteria directly.
